# Exercise MRI stress testing of the human heart at 3 Tesla: measurement precision of biventricular function and aortic blood flow during steady-state bicycling exercise

**DOI:** 10.1007/s10334-025-01304-9

**Published:** 2025-12-04

**Authors:** Hugo Klarenberg, Martijn Froeling, Tim Leiner, Hildo J. Lamb, S. Matthijs Boekholdt, Harald T. Jørstad, Gustav J. Strijkers, Adrianus J. Bakermans

**Affiliations:** 1 Biomedical Engineering and Physics, Amsterdam University Medical Center, University of Amsterdam, Meibergdreef 9, 1105 AZ Amsterdam, The Netherlands; 2 Department of Radiology, University Medical Center Utrecht, Utrecht, The Netherlands; 3 Department of Radiology, Mayo Clinic, Rochester, MN USA; 4 Department of Radiology, Leiden University Medical Center, Leiden, The Netherlands; 5 Department of Cardiology, Amsterdam University Medical Center, University of Amsterdam, Amsterdam, The Netherlands; 6 Department of Radiology and Nuclear Medicine, Amsterdam University Medical Center, University of Amsterdam, Amsterdam, The Netherlands

**Keywords:** Accelerated MRI, Athlete’s heart, Ergometry, Exercise intolerance, HFpEF

## Abstract

**Objective:**

This work aimed to demonstrate the feasibility of quantifying heart function during bicycling exercise with dynamic real-time cine MRI at 3 Tesla, and to assess its measurement precision.

**Materials and methods:**

Twelve volunteers performed steady-state bicycling exercise, while real-time cine MR images were collected using a 72-channel receiver coil array and a parallel imaging acceleration factor of 5. Biventricular end-diastolic and end-systolic (ESV) volumes and function during exercise were compared with resting-state real-time cine MRI and conventional cardiac-gated cine MRI under breath holding, and validated against 2D phase-contrast MRI-based estimates of aortic blood flow. Precision was evaluated as the inter-session measurement repeatability.

**Results:**

Left (LV) and right ventricular (RV) stroke volumes (SV) increased progressively with exercise intensity, which was mediated by a decrease in ESV. Likewise, LV SV estimated with 2D phase-contrast MRI increased from 90 ± 17 mL at rest to 114 ± 29 mL during vigorous-intensity exercise. Repeatability coefficients were 52% and 41% for LV SV at moderate- and vigorous-intensity exercise, while RV SV repeatability coefficients were 58% and 42%, respectively.

**Discussion:**

We established an exercise MRI stress testing protocol for quantifying biventricular volumes and function during moderate- and vigorous-intensity steady-state bicycling exercise.

**Supplementary Information:**

The online version contains supplementary material available at 10.1007/s10334-025-01304-9.

## Introduction

The ability to perform physical exercise is an important clinical indicator of physical health [1]. As such, cardiopulmonary exercise stress testing (CPET) is integrated into the workflow of the cardiology clinic, and crucially informs on the differential contributions of the skeletal muscle, cardiovascular and pulmonary systems to impaired exercise tolerance experienced by patients in their daily lives [2]. Moreover, such functional exercise testing can aid diagnosis and risk stratification in otherwise notoriously difficult-to-diagnose multifactorial diseases, such as heart failure with preserved ejection fraction (HFpEF) [3, 4]. While transthoracic echocardiography is used abundantly in the cardiology clinic for noninvasively evaluating cardiac performance, its application during exercise is hampered by deteriorating image quality, a strong operator dependency, and generally poor visibility of the right ventricle (RV). With a growing recognition of the importance of RV function and the pulmonary circulation in exercise (in)tolerance [5], robust and reliable imaging of both ventricles during exercise is highly desired.

Magnetic resonance imaging (MRI) is a reliable technique for assessing biventricular function and morphology at rest. Research interest and development efforts for generating protocols that enable meaningful MRI during exercise are increasing [6]. Indeed, the inclusion of exercise MRI stress testing of the heart in guidelines for standardized cardiovascular MRI protocols [7] suggests that the approach has matured into common methodology. Yet, exercise MR stress testing still comes with many challenges, particularly related to obtaining sufficient temporal resolution to quantify cardiac performance at high heart rates [8], mitigating exercise- and respiratory-induced motion artifacts, and quantifying functional measures in a meaningful way. An often-used approach is ‘real-time’ cine MRI [9], i.e., acquiring images that capture the exercise dynamics as they occur, without the need for repetition or physiology gating [10] that is common with conventional breath-hold resting-state MRI of the heart [7]. The impact of motion can be reduced by instructing the subject to cease exercise [11–13] and even hold their breath [14, 15] for immediate post-exercise imaging. Alternatively, retrospective identification and sorting of real-time cine MR images pertaining to end-diastolic and end-systolic cardiac phases as well as a consistent respiratory phase has been done visually [9, 16], or by using simultaneous recordings of the ECG-signal and respiration [17]. Algorithms that help sort real-time images into consistent cardiac and respiratory phases [18] may reduce the required processing time and effort while improving quantification accuracy and precision. Until now, comprehensive measurement repeatability evaluations of exercise MRI stress testing of the heart are relatively scarce [19, 20]. Moreover, most exercise studies were performed at 1.5 Tesla [9, 11, 13, 14, 17–19], focused on the left ventricle (LV) only [11, 13, 15, 18], and were conducted at relatively low exercise intensities [12, 15, 18, 20].

Here, we report on the feasibility of quantifying biventricular function during vigorous-intensity bicycling exercise using real-time MRI at 3 Tesla with a parallel imaging acceleration factor of 5 facilitated by a 72-channel receiver coil array. We introduce an algorithm for retrospective alignment of real-time acquired images into corresponding respiratory and cardiac phases for volumetric quantifications. We hypothesized that this approach would allow capturing how exercise-induced augmentation of myocardial contractility [21] through reduced end-systolic volumes (ESV) increases stroke volumes (SV) of both ventricles. Measurement precision of this exercise MRI stress testing protocol was established in volunteers, and quantitative measures of heart function were compared against 2D phase-contrast (PC) MRI-based estimates of blood flow through the ascending aorta at rest and during exercise.

## Methods

This feasibility study in healthy volunteers was approved by the local institutional review board (NL71689.018.20; Amsterdam University Medical Center, Amsterdam, The Netherlands). All subjects provided written informed consent before inclusion in the study. We recruited 12 (male/female, 6/6) adult volunteers, all non-smoking and without a history of cardiovascular disease. Exclusion criteria were physical limitations for performing supine bicycling exercise, claustrophobia, and contraindications for MR examination at 3 Tesla.

### MRI protocol

All examinations were performed with a 3 Tesla MR system (bore diameter, 70 cm; Ingenia; Philips, Best, The Netherlands) equipped with a custom-built 72-channel anterior receiver coil array [22] and a 12-channel posterior receiver coil array integrated in the patient table. An MR-compatible bicycle ergometer (MR Pedal; Lode BV, Groningen, The Netherlands) was mounted on the foot-end of the patient table and interfaced to a personal computer for remote adjustment of bicycling workload and monitoring of bicycling revolutions per minute (Lode Ergometry Manager 10; Lode BV). Subjects were positioned supine and connected to a 4-lead electrocardiography sensor (ECG; Invivo, Philips) and a peripheral pulse unit (PPU; Invivo, Philips) placed on the right index finger for heart rate monitoring and gated MR acquisitions.

After isocenter positioning and scout imaging, a stack of retrospectively ECG-gated short-axis çine 2D image series covering the whole heart was acquired over 2 breath holds at end-expiration with a Cartesian sampled balanced steady-state free precession (bSSFP) sequence [23]. Imaging parameters: field of view, 350 × 350 mm^2^; slice thickness/gap, 8/1 mm; repetition time (TR)/echo time (TE), 2.8/1.4 ms; flip angle, 45°; parallel imaging (SENSE, sensitivity encoding) acceleration factor, 6; matrix, 124 × 124; reconstructed in-plane resolution, 1 × 1 mm; reconstructed cardiac phases, 30; number of slices, 15. Next, a series of non-gated real-time short-axis cine MR images were acquired using a Cartesian sampled bSSFP sequence with imaging parameters that were empirically determined with a focus on achieving a high temporal resolution while preserving sufficient contrast for image segmentation-based volumetry: field of view, 352 × 288 mm^2^; slice thickness (no gap), 10 mm; sequential slice acquisition; TR/TE, 3.0/1.45 ms; flip angle, 55°; SENSE factor, 5; partial Fourier factor, 0.7; matrix, 88 × 72; reconstructed in-plane resolution, 2 × 2 mm^2^; temporal resolution, 33 ms. A series of real-time cine MRI with 40 frames/slice × 15 slices was acquired during 1 end-expiration breath hold of approximately 20 s, and was used for the validation of quantitative volumetry based on real-time cine MRI against conventional ECG-gated cine MRI. Then, another series of real-time cine MRI was acquired during free breathing for 10 s/slice (i.e., 300 frames/slice) to cover multiple respiratory cycles per slice in a stack of 15–17 consecutive slices that covered the whole heart while allowing for cardiac displacement due to respiration. To compare stroke volume (SV) quantifications based on cine MRI with measurements of blood flow through the aorta, a peripheral pulse-gated free-breathing 2D PC-MRI scan was acquired orthogonally to the ascending and descending aorta for the quantification of through-plane aortic blood flow velocity. Imaging parameters: field of view, 350 × 350 mm^2^; slice thickness, 8 mm; TR/TE, 4.4/2.8 ms; flip angle, 10°; SENSE factor, 4; matrix, 140 × 121; reconstructed in-plane resolution, 1.22 × 1.22 mm^2^; 30 frames/*R*–*R* interval; velocity encoding (*V*_enc_), 150 cm/s.

After these resting-state measurements, the subject’s feet were secured in the ergometer pedals with Velcro straps for an in-magnet bicycling exercise protocol [24]. Subjects were instructed to commence bicycling at a cadence of approximately 70 revolutions per minute, while keeping torso movement to a minimum by holding handles along both sides. Each subject’s age-dependent supine maximal heart rate was estimated via *HR*_max_ [beats/min] = 211 − 0.64 [beats/min/years] × age [years] [25], and multiplied by 0.9 to adjust for the lower *HR*_max_ during supine vs. upright bicycling ergometry [26]. By remotely adjusting the bicycling workload according to normative values for supine bicycling ergometry [27], exercise intensity was targeted first at steady-state exercise at 60% of supine *HR*_max_ (moderate intensity), followed by ramped-incremental exercise up to steady-state exercise at 80% of supine *HR*_max_ (vigorous). Both steady-state exercise stages were maintained for at least 5 min, during which a coil sensitivity scan for SENSE acceleration was re-acquired, followed by a stack of real-time cine MRI series of the whole heart (300 frames/slice, 15–17 slices) and a 2D PC-MRI scan of the aorta (*V*_enc_, 200 cm/s at moderate-intensity exercise; *V*_enc_, 250 cm/s at vigorous-intensity exercise), all under free-breathing conditions.

We assessed inter-session repeatability of our measurements in a subset of 6 volunteers (male/female, 3/3), who repeated the same protocol on a separate day within 1 week.

### MR data analyses

To avoid introducing any inter-reader variability in our measurement repeatability assessments, all analyses were done by one reader (HK, 4 years of experience) under the supervision of an experienced investigator (AJB, 16 years of experience). Left ventricular (LV) and right ventricular (RV) volumes at end-diastole (EDV) and end-systole (ESV) were estimated from the short-axis cine MRI series by semi-automatically contouring the endocardial chamber walls in Medis Suite 4.0 (QMass 8.1; Medis medical imaging systems BV, Leiden, The Netherlands), and used to derive LV and RV SV, ejection fraction (EF), and cardiac output (CO). LV and RV trabecular tissue and papillary muscles were included in the blood pool volumes. Automatically generated contours were carefully reviewed, and corrected manually where needed, e.g., in case of artifacts due to blood flow or due to changes in coil sensitivity profiles during free breathing with bicycling exercise. LV mass was estimated by segmenting the epicardial LV wall, subtracting the LV chamber volume, and multiplying the volume by the myocardial specific density (1.05 g/mL) [28].

**Fig. 1:**
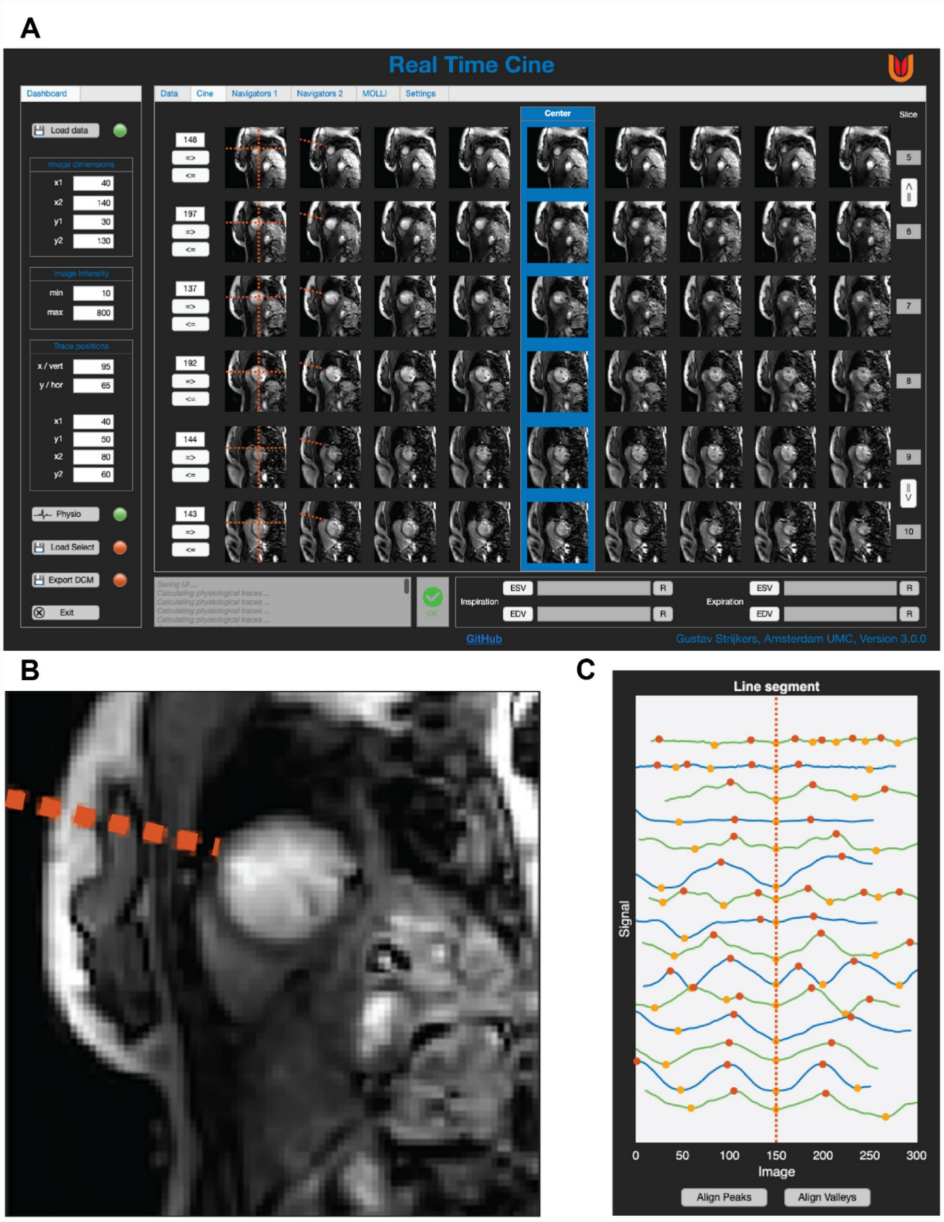
Screenshot of MATLAB-based user interface for retrospective frame sorting (**A**). A ‘navigator’ line (red line) is defined manually over the chest-lung interface (**B**). The mean signal intensity over this line is then plotted against time (**C**), with detection of peaks (orange dots) and valleys (ocher dots) that reflect end-expiration and end-inspiration phases, respectively. Each trace (300 frames; 10 s) represents a slice in the stack of real-time cine MRI series acquired from apex (top) to base (bottom) during free breathing. Respiratory rate in this example of moderate-intensity bicycling exercise was approximately 15 breaths/minute (i.e., 2.5 respiratory cycles/trace), although some variations in breathing frequency occurred. Respiratory phases could not be visualized clearly in more apical slices due to less motion of the chest or abdomen in these images, resulting in low or absent signal intensity peaks or valleys. Buttons allow the user to respiratory phase-align all slices based on detected peaks or valleys, retrospectively synchronizing respiration between consecutively acquired slices in the stack of real-time cine MRI series. Then, the user can define the end-systolic and end-diastolic phase per slice by positioning the corresponding frame in the ‘center’ (blue box). The resulting images are aligned to both the cardiac as well as the respiratory phases, and can be exported in their original DICOM format for volumetric analyses. The software is available on GitHub (https://github.com/Moby1971/RealTimeCine-v3.0)

Unlike conventional cardiac-gated cine MRI series acquired under breath holding, real-time cine MRI series acquired during free breathing do not have their slices aligned to cardiac and respiratory phases. This misalignment must be accounted for in volumetric quantifications, and complicates a straightforward evaluation of cardiac performance under free-breathing conditions. To facilitate the identification of end-diastolic and end-systolic cardiac phases at a specific respiratory phase in each slice, we developed a MATLAB-based (The MathWorks, Inc., Natick, MA, USA) processing algorithm and user interface (Fig. 1) to assist in retrospective frame sorting as follows. The software is available on GitHub (https://github.com/Moby1971/RealTimeCine-v3.0). First, a respiratory ‘navigator’ line is manually placed over the diaphragm or across the chest muscle, targeting the distinct contrast at the liver–lung or chest–lung interface. The mean signal intensity of this line is then plotted over time for each slice, capturing the respiratory cycle [18]. Phases of end-expiration (local maximum signal intensity) and end-inspiration (local minimum signal intensity) are then detected by identifying peaks and valleys in this signal intensity time curve, with these frames highlighted to assist the user. The software can then automatically align all slices to either the end-expiration or end-inspiration phase, effectively adjusting the reels of each slice in the stack of real-time cine MRI series relative to one another. Subsequently, the user manually identifies the end-diastolic (maximal chamber blood pool) and end-systolic (minimal chamber blood pool) for each slice, within a proximity window (−7 to +7 frames/462 ms at rest; −5 to +5 frames/330 ms during exercise) around the established respiratory phase. The process results in a stack of images aligned for both the cardiac as well as the respiratory phases, covering the whole heart at end-diastole and end-systole during both end-expiration and end-inspiration. These images can then be exported in their original DICOM format for analysis in Medis Suite 4.0 (QMass 8.1; Medis medical imaging systems BV) as described above. Ventricular volumes were quantified for ECG-gated cine MRI series and real-time cine MRI series during breath holding, real-time cine MRI series at end-expiration and at end-inspiration during free breathing, and real-time cine MRI series at end-expiration during free breathing with bicycling exercise.

Blood flow through the aorta was quantified using 2D PC-MRI scans (QFlow 8.1; Medis medical imaging systems BV). Regions of interest (ROIs) were identified for the ascending (aAo) and descending aorta (dAo) in the magnitude image, propagated throughout the cardiac cycle, and adjusted manually where needed. Forward and backward flow volumes (mL) through each ROI were calculated over the cardiac cycle [28]. The ratio of net flow through the descending aorta to the ascending aorta (dAo/aAo) was used to assess blood flow distribution, with higher values indicating a relatively larger proportion of flow directed to the lower body.

### Statistical analyses

Data are presented as mean ± standard deviation (SD). Differences in outcome parameters (i.e., LV and RV EDV and ESV, SV, EF, CO, aAo and dAo flow, dAo/aAo net flow ratio) between different sequences (ECG-gated vs. real-time cine MRI) and between respiratory conditions (end-expiration vs. end-inspiration during free breathing, and end-expiration breath holding vs. end-expiration during free breathing) were examined using pair-wise comparisons (two-sided paired *t*-tests). Effects of bicycling exercise (rest vs. moderate-intensity exercise vs. vigorous-intensity exercise) were analyzed using repeated-measures analysis of variance (ANOVA). The statistical significance level was set to *p* < 0.05, and *p*-values were adjusted for multiple comparisons by controlling the false discovery rate as proposed by Benjamini and Hochberg [29]. Limits of agreement between ECG-gated cine-MRI and real-time cine MRI volumetry, as well as between real-time cine MRI-based quantifications of LV SV and 2D PC-MRI measurements of aAo forward flow were calculated as the mean difference between the two metrics (i.e., bias) ± 1.96 times the SD of the difference between the two measurements (i.e., limits of agreement) in (mL) [30]. Correlations between measurement methods were tested with Pearson’s correlation analyses. Bland–Altman analyses were used to assess inter-session measurement repeatability [30], with the coefficient of repeatability calculated as 1.96 times the SD of the differences between repeated measurements. As such, a lower coefficient of repeatability reflects narrower confidence intervals and a higher measurement precision. This measure of precision is expressed as a percentage of the group mean value for that condition. Test–retest intraclass correlation coefficients were estimated based on a single-measurement absolute-agreement two-way mixed-effects model [31]. Analyses were performed using R (version 4.2.2, The R Foundation for Statistical Computing, Vienna, Austria) and Rstudio (2022.07.2 + 576; Posit, Boston, MA).

## Results

Twelve participants (male/female, 6/6; age, 28.8 ± 2.7 years; weight, 68.4 ± 9.4 kg; height, 173 ± 7 cm; body mass index, 22.6 ± 1.7 kg/m^2^) completed the protocols. Quantitative volumetric and functional results for these subjects under various conditions are reported in Table 1. In six of our volunteers (male/female, 3/3), all measurements were repeated on a separate day for an evaluation of inter-session measurement repeatability.

**Table 1:** Quantitative exercise MRI stress testing of biventricular volumes and function in *n* = 12 volunteers at 3 Tesla

Exercise intensity	Rest				Moderate	Vigorous
Workload (W)					68 ± 16	123 ± 22
Heart rate (beats/min)	56 ± 6				105 ± 3	136 ± 8
% of maximal heart rate	33 ± 3				61 ± 2	78 ± 4
*Volumetry (cine MRI)*						
Cine MRI approach	ECG-gated	real-time				
Breath holds (*n*)	2	1	none (free breathing)			
Respiratory phase	Expiration	Expiration	Inspiration	Expiration	Expiration	Expiration
LV EDV (mL)	170 ± 28	158 ± 28*	155 ± 28	155 ± 26	164 ± 27	166 ± 36
LV ESV (mL)	72 ± 12	71 ± 21	65 ± 15	71 ± 20	62 ± 15	50 ± 18^b^
LV SV (mL)	99 ± 17	86 ± 14*	90 ± 16	84 ± 18	102 ± 17^a^	116 ± 23^b^
LV EF (%)	58 ± 3	55 ± 6	58 ± 5	54 ± 9	62 ± 6^a^	71 ± 6^b^
LV CO (L/min)	5.5 ± 1.2	4.9 ± 1.1	5.1 ± 1.1	4.7 ± 1.3	10.7 ± 1.7^c^	15.8 ± 3.6^c^
LV mass (g)	150 ± 38	151 ± 32	154 ± 35	157 ± 37	152 ± 36	154 ± 38
RV EDV (mL)	172 ± 28	156 ± 26**	161 ± 29	156 ± 26	159 ± 31	159 ± 34
RV ESV (mL)	80 ± 16	75 ± 18	77 ± 17	80 ± 18	66 ± 20^b^	56 ± 21^b^
RV SV (mL)	92 ± 18	81 ± 14*	84 ± 19	75 ± 18	94 ± 18	104 ± 17^a^
RV EF (%)	54 ± 5	52 ± 7	52 ± 7	48 ± 8	59 ± 7^b^	66 ± 7
RV CO (L/min)	5.2 ± 1.2	4.5 ± 1.0	4.7 ± 1.2	4.3 ± 1.3	9.8 ± 1.8^c^	14.1 ± 3.0^c^
*Aortic blood flow (2D phase-contrast MRI)*						
aAo flow (mL)				90 ± 17	106 ± 17	114 ± 29
dAo flow (mL)				56 ± 11	76 ± 12	78 ± 23
d/a flow ratio (–)				0.61 ± 0.10	0.74 ± 0.10^a^	0.71 ± 0.17

Data are presented as mean ± standard deviation. *aAo* Ascending aorta, *CO* Cardiac output, *dAo* Descending aorta, *EDV* End-diastolic volume, *EF* Ejection fraction, *ESV* End-systolic volume, *LV* Left ventricle, *RV* Right ventricle, *SV* Stroke volume. *p < 0.05, **p < 0.01 compared to conventional ECG-gated cine MRI; ^a^*p* < 0.05, ^b^*p* < 0.01, ^c^*p* < 0.001 compared to rest

### Real-time cine MRI vs. ECG-gated cine MRI estimates of ventricular volumes and function

**Fig. 2:**
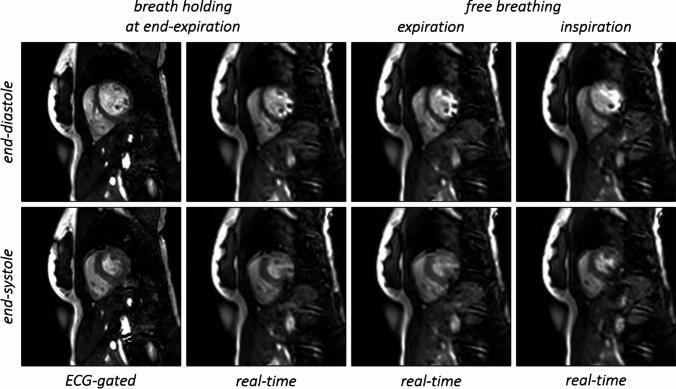
Real-time cine MRI at 3 Tesla. The left column shows equatorial short-axis views at end-diastole (top) and end-systole (bottom) acquired with conventional ECG-gated cine MRI at rest during breath holding at end-expiration. For comparison, real-time cine MRI series were obtained during breath holding (second column), and during free breathing, from which end-expiration (third column) and end-inspiration (right column) phases were retrospectively sorted

**Fig. 3:**
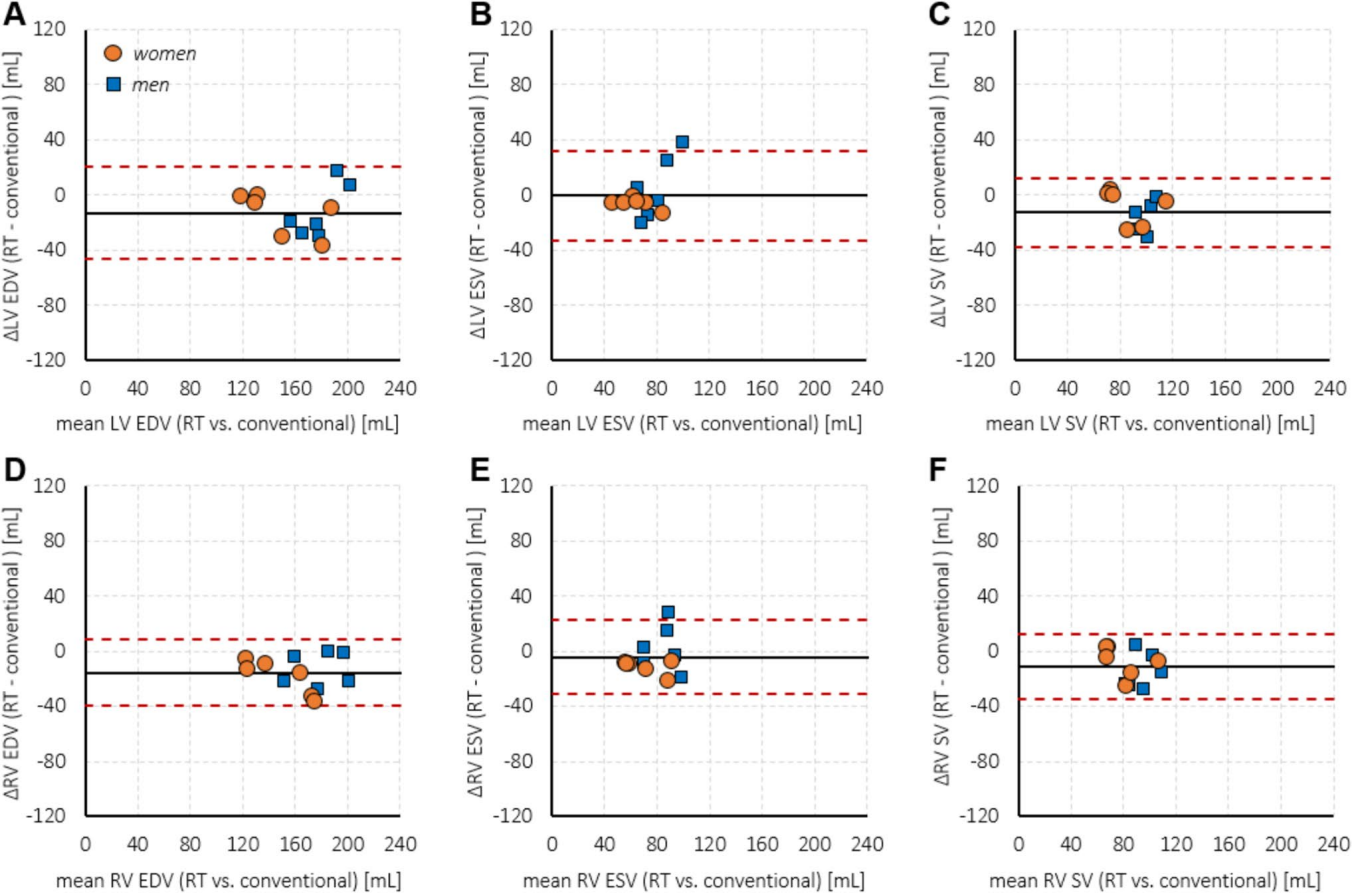
Bland–Altman analyses comparing left ventricular (LV) end-diastolic volume (EDV; **A**), LV end-systolic volume (ESV; **B**), and LV stroke volume (SV; **C**), as well as right ventricular (RV) EDV (**D**), RV ESV (**E**), and RV SV (**F**) measured using real-time (RT) cine MRI against conventional ECG-gated cine MRI in healthy volunteers (*n* = 12; male/female, 6/6) during breath holding at rest. The dashed red lines indicate the limits of agreement around the bias (black solid line) between the two methods

Quantifications of LV and RV EDV and ESV based on real-time cine MRI were validated against those obtained from conventional ECG-gated cine MRI, with both stacks acquired at rest during breath holding (Fig. 2). Bland–Altman analyses are shown in Fig. 3. This direct comparison between the two approaches revealed a bias ± limits of agreement of −13 ± 33 mL (−8 ± 9%; *p* = 0.045) for measuring LV EDV, with a mean value of 164 ± 27 mL, and a bias ± limits of agreement of −16 ± 24 mL (−9 ± 6%; *p* = 0.008) for measuring RV EDV, with a mean value of 164 ± 26 mL. These data indicate that real-time cine MRI volumetry underestimates EDV relative to ECG-gated cine MRI. Yet, LV ESV (0 ± 33 mL; *p* = 0.999) and RV ESV (−4 ± 27 mL; *p* = 0.650) were similar for both methods. Consequently, real-time cine MRI volumetry underestimated LV SV (−12 ± 25 mL; −13 ± 12%; *p* = 0.036) and RV SV (−11 ± 24 mL; −12 ± 13%; *p* = 0.026). LV mass was similar for both methods (2 ± 19 g; *p* = 0.822).

### Impact of respiration on chamber volumes and function

Real-time cine MRI enabled us to quantify LV and RV volumes and function at end-expiration and end-inspiration during free breathing, allowing a comparison between respiratory phases. Figure 2 shows real-time cine MR images acquired during free breathing and under breath holding. We did not detect any differences in any of the volumetric or functional parameters between end-expiration and end-inspiration during free breathing (Table 1). Moreover, parameters at end-expiration during free breathing were similar to those during a breath hold at end-expiration.

### Cardiac function during steady-state exercise

Participants performed in-magnet supine bicycling exercise at a steady-state heart rate of 105 ± 3 beats/min against a workload of 68 ± 16 W during moderate-intensity exercise (i.e., 61 ± 2% of their predicted *HR*_max_), and at 136 ± 8 beats/min against 123 ± 22 W during vigorous-intensity exercise (78 ± 4% of *HR*_max_). Measurements during vigorous exercise were missed in three subjects, due to a coil connection failure, operator error, or subject displacement that shifted their heart out of the field of view.

**Fig. 4:**
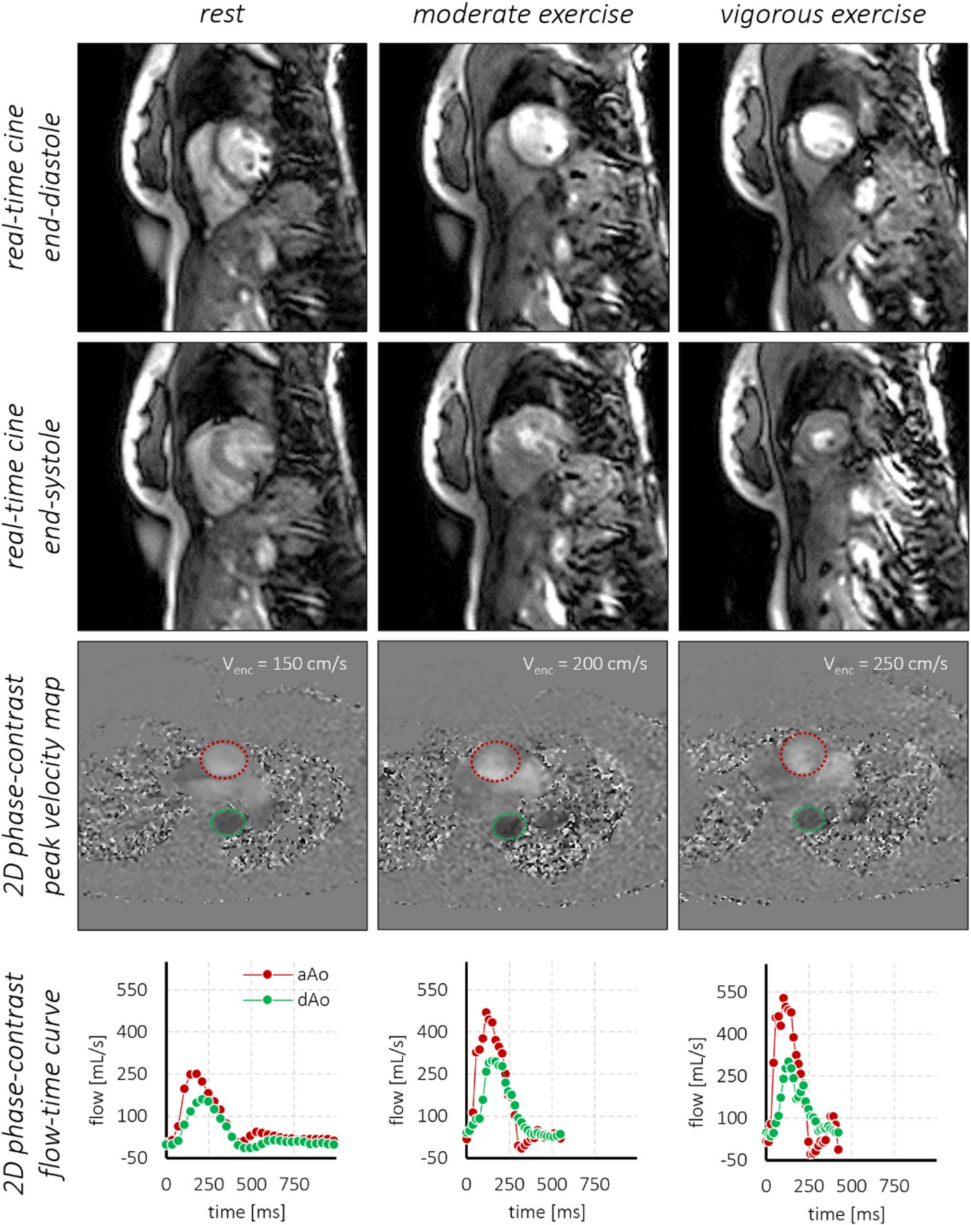
Exercise MRI stress testing in a female volunteer (age, 30 years). Real-time cine MRI at 3 Tesla was used to acquire a stack of short-axis cine MRI series at rest (heart rate, 58 beats/min), during steady-state moderate-intensity bicycling exercise (workload, 60 W; heart rate, 105 beats/min), and during steady-state vigorous-intensity bicycling exercise (workload, 115 W; heart rate, 136 beats/min). Cine MRI series were used for volumetric quantifications, and 2D phase-contrast MRI was used to quantify blood flow through the aorta. Series are available as videos in the Online Supplementary Information

Figure 4 shows real-time cine MR images acquired during exercise. Series are available as videos in the Online Supplementary Information. During moderate exercise (Table 1), LV SV increased to 102 ± 17 mL, which is 21 ± 35% higher than at rest (*p* = 0.039). This increase seemed to be primarily mediated by LV ESV (−13 ± 22%; *p* = 0.072) rather than LV EDV (+5 ± 25%; *p* = 0.302). As a result, LV CO more than doubled to 10.7 ± 4.7 L/min (*p* < 0.001). Similarly, RV SV was 94 ± 18 mL during moderate exercise (+ 24 ± 40%; *p* = 0.051), with RV ESV decreasing by 18 ± 16% (*p* = 0.008), while RV EDV did not change (+ 2 ± 15%; *p* = 0.731). RV CO was 9.8 ± 1.8 L/min. With vigorous exercise (Table 1), LV and RV SV were 116 ± 23 mL (+ 10 ± 13% vs. moderate exercise; *p* = 0.050) and 104 ± 17 mL (+ 8 ± 18%; *p* = 0.197), respectively. Again, this was mainly due to a further reduction in ESV (−20 ± 20%; *p* = 0.014 for the LV, and −20 ± 20%; *p* = 0.016 for the RV), while EDV did not change (−1 ± 10%; *p* = 0.770 for the LV, and −4 ± 15%; *p* = 0.731 for the RV). Vigorous exercise led to a nearly threefold increase in CO compared to rest (*p* < 0.001), with LV CO reaching 15.8 ± 3.6 L/min and RV CO reaching 14.1 ± 3.0 L/min.

**Fig. 5:**
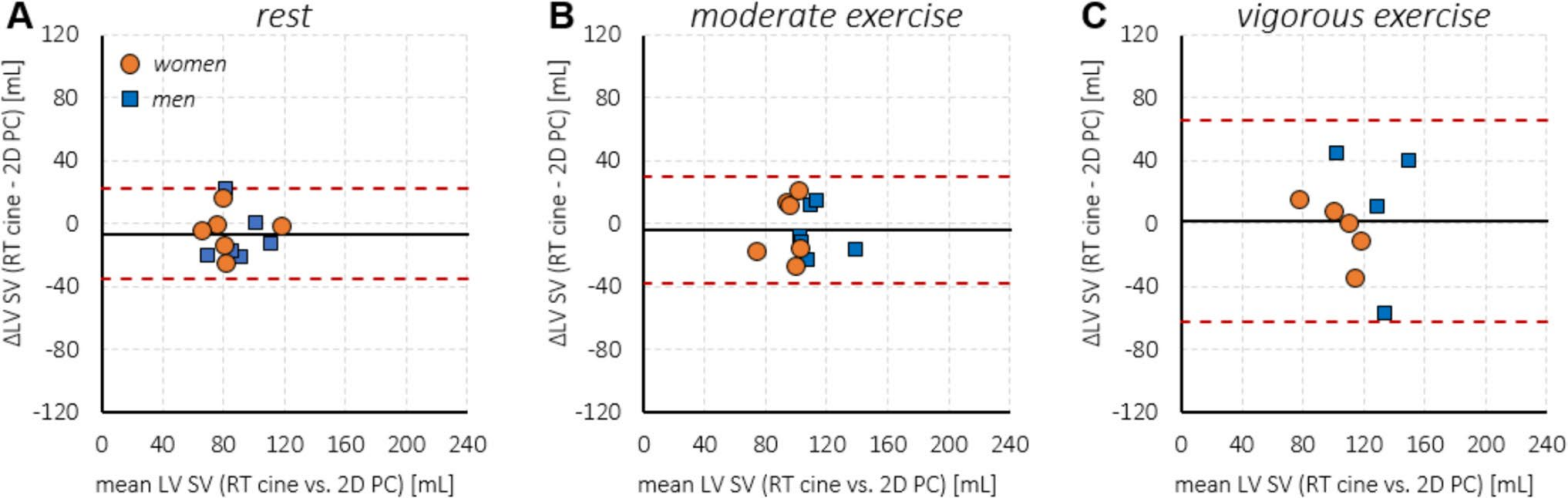
Bland–Altman analyses comparing left ventricular (LV) stroke volume (SV) measured using real-time (RT) cine MRI against estimates derived from 2D phase-contrast (PC) MRI of the forward blood flow through the ascending aorta in healthy volunteers (*n* = 12; male/female, 6/6) at rest (**A**), during steady-state moderate-intensity bicycling exercise (**B**), and during steady-state vigorous-intensity bicycling exercise (**C**). The dashed red lines indicate the limits of agreement around the bias (black solid line) between the two methods

Volumetric measurements from real-time cine MRI were validated against quantifications of blood flow through the aorta using 2D PC-MRI (Fig. 4). Series are available as videos in the Online Supplementary Information. The aAo forward flow was 90 ± 17 mL at rest, increased to 106 ± 17 mL (+ 17 ± 17%; *p* = 0.010 vs. rest) during moderate exercise, and was 114 ± 29 mL (+ 6 ± 21%; *p* = 0.414 vs. moderate exercise) during vigorous exercise. Bicycling exercise also increased the proportion of blood flow directed to the lower body, as evidenced by the increase in the dAo/aAo net flow ratio from 0.61 ± 0.10 at rest to 0.74 ± 0.1 during moderate exercise (*p* = 0.049 vs. rest) and 0.71 ± 0.17 during vigorous exercise (*p* = 0.686 vs. moderate exercise). We compared LV SV from real-time cine MRI with aAo forward flow in Bland–Altman analyses across all stages of the exercise protocol (Fig. 5). The bias ± limits of agreement were −7 ± 29 mL at a mean LV SV of 87 ± 16 mL at rest, −4 ± 34 mL at a mean of 104 ± 15 mL during moderate exercise, and + 2 ± 64 mL at a mean of 115 ± 21 mL during vigorous exercise. While the two methods correlated moderately at rest (*r* = 0.64; *p* = 0.024), this correlation weakened with increasing exercise intensity (*r* = 0.50; *p* = 0.095 for moderate exercise, and *r* = 0.25; *p* = 0.522 for vigorous exercise).

### Repeatability of exercise MRI stress testing

Six volunteers (male/female, 3/3) repeated the protocol on a separate day within 1 week. Their heart rates were similar for both visits at rest (56 ± 7 beats/min vs. 57 ± 6 beats/min; *p* = 0.805), and they required similar bicycling workloads (69 ± 13 W vs. 71 ± 12 W; *p* = 0.528) to achieve similar heart rates during steady-state moderate exercise (104 ± 2 beats/min vs. 104 ± 3 beats/min; *p* = 0.844). Likewise, workloads (116 ± 11 W vs. 121 ± 14 W; *p* = 0.293) for steady-state vigorous exercise (134 ± 8 beats/min vs. 134 ± 4 beats/min; *p* = 1.0) were similar for both sessions. As such, the steady-state exercise protocol was highly repeatable and induced a consistent exercise intensity regime across sessions.

**Fig. 6:**
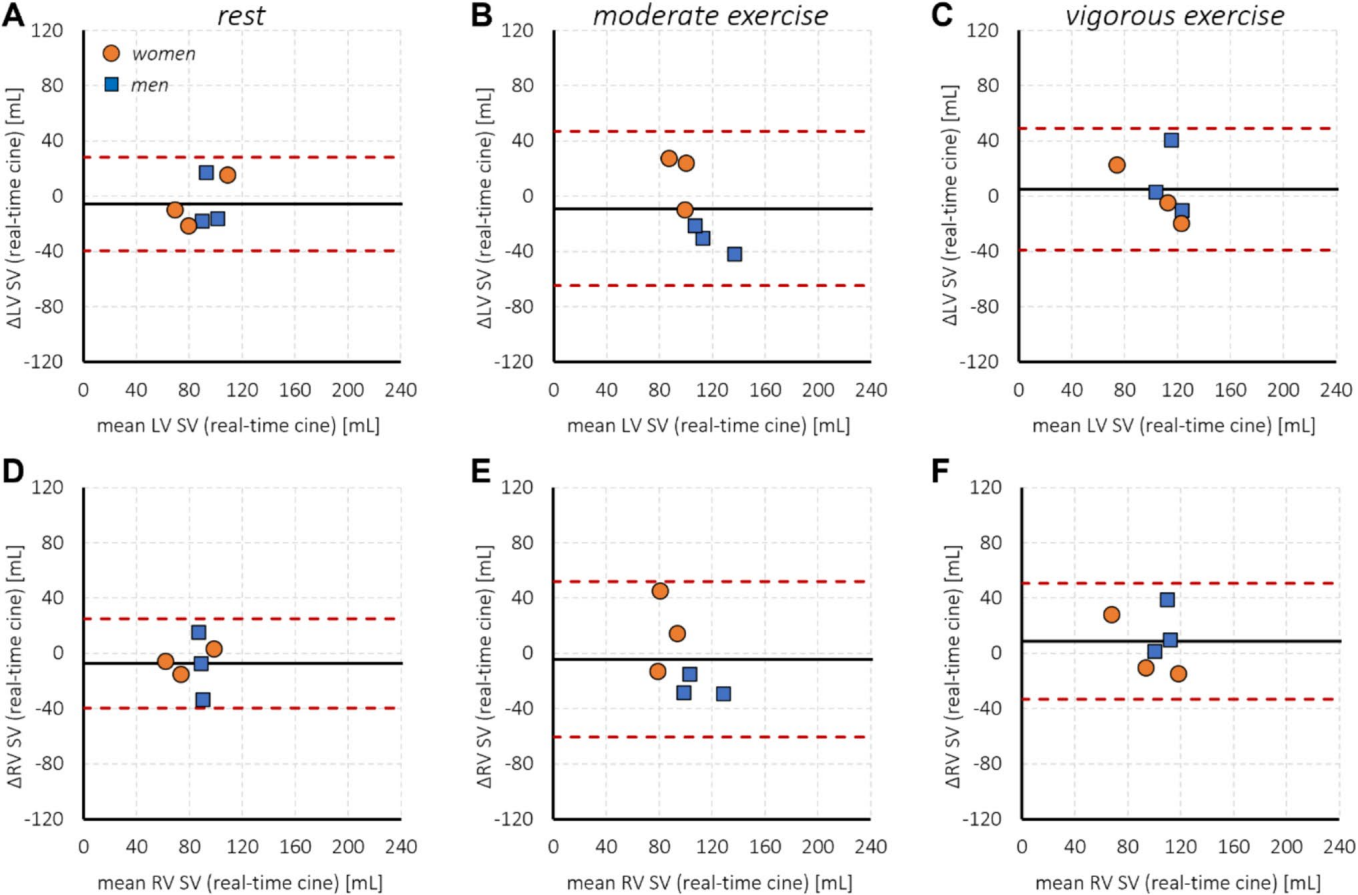
Precision of exercise MRI stress testing. Bland–Altman analyses of inter-session measurement repeatability of left ventricular (LV) and right ventricular (RV) stroke volume (SV) using real-time cine MRI at 3 Tesla at rest (**A**, **D**), during steady-state moderate-intensity bicycling exercise (**B**, **E**), and during steady-state vigorous-intensity bicycling exercise (**C**, **F**) in healthy volunteers (*n* = 6 × 2 repeated sessions; male/female, 3/3). Dashed red lines represent the 95% confidence interval at 1.96 times the standard deviation of the differences between the two measurements around the mean difference between consecutive sessions (black solid line). Details on these parametric 95% confidence intervals are available in the Online Supplementary Information

The inter-session repeatability coefficient for LV SV measurements using real-time cine MRI (Fig. 6) was 37% at rest, with a mean LV SV of 90 ± 14 mL and a mean difference of −5 ± 17 mL between sessions. During moderate-intensity exercise, the repeatability coefficient increased to 52%, with a mean of 107 ± 17 mL and a mean difference of −9 ± 28 mL. For vigorous-intensity exercise, the repeatability coefficient was 41%, with a mean of 109 ± 18 mL and a mean difference of + 5 ± 23 mL.

For RV SV, the repeatability of real-time cine MRI measurements was very similar (Fig. 6). At rest, the inter-session repeatability coefficient was 39%, with a mean RV SV of 83 ± 13 mL and a mean difference of −7 ± 16 mL between sessions. The repeatability coefficient increased to 58% during moderate exercise, with a mean of 97 ± 18 mL and a mean difference of −4 ± 29 mL. During vigorous exercise, the repeatability coefficient was 42%, with a mean of 100 ± 18 mL and a mean difference of 9 ± 21 mL.

Using 2D PC-MRI of aAo forward flow to estimate LV SV, the inter-session repeatability of LV SV measurements was 35% at rest, with a mean aAo forward flow of 91 ± 15 mL and a mean difference of 3 ± 16 mL between sessions. During moderate exercise, the repeatability coefficient improved to 22%, with a mean of 110 ± 13 mL and a mean difference of −13 ± 12 mL. The repeatability coefficient was 47% during vigorous exercise, with a mean of 121 ± 31 mL and a mean difference of −9 ± 28 mL.

Inter-session repeatability estimates for all volumetric and functional parameters are summarized in Table 2. Details on the parametric 95% confidence intervals are available in the Online Supplementary Information. Intraclass correlation coefficients ranged from 0 for some parameters with low between-subject variance (e.g., LV EF) up to >0.8 for LV EDV and RV EDV (Supplemental Table 2). Our results indicate that measurement precision ranges from approximately 40% to 60%, with only a moderate decline in precision during steady-state bicycling exercise compared to resting conditions.

**Table 2:** Inter-session measurement repeatability of exercise MRI stress testing in *n* = 6 volunteers

	Rest			Moderate exercise			Vigorous exercise		
	Mean	Bias	CR (%)	Mean	Bias	CR (%)	Mean	Bias	CR (%)
LV EDV (mL)	156 ± 20	−7 ± 8	9	165 ± 22	−6 ± 25	30	158 ± 25	−13 ± 13	16
LV ESV (mL)	66 ± 7	−1 ± 15	45	57 ± 8	3 ± 12	41	49 ± 10	−18 ± 13	50
LV SV (mL)	90 ± 14	−5 ± 17	37	107 ± 17	−9 ± 28	52	109 ± 18	5 ± 23	41
LV EF (%)	58 ± 3	−1 ± 10	33	65 ± 3	−2 ± 9	27	69 ± 4	10 ± 10	28
LV CO (L/min)	5.1 ± 1.0	−0.3 ± 1.5	57	11.1 ± 1.7	−0.9 ± 3.1	55	14.6 ± 2.3	0.7 ± 3.7	49
RV EDV (mL)	156 ± 18	5 ± 13	16	160 ± 26	11 ± 27	33	158 ± 35	8 ± 21	27
RV ESV (mL)	73 ± 14	12 ± 17	46	62 ± 16	16 ± 9	29	58 ± 18	−1 ± 19	66
RV SV (mL)	83 ± 13	−7 ± 17	39	97 ± 18	−4 ± 29	58	100 ± 18	9 ± 21	42
RV EF (%)	53 ± 7	−6 ± 9	34	61 ± 6	−6 ± 9	28	64 ± 4	5 ± 10	31
RV CO (L/min)	4.7 ± 0.8	−0.5 ± 1.1	46	10.1 ± 1.9	−0.4 ± 3.0	59	13.5 ± 2.6	1.2 ± 3.3	48
aAo flow (mL)	91 ± 15	3 ± 16	35	110 ± 13	−13 ± 12	22	121 ± 31	−9 ± 28	46
dAo flow (mL)	57 ± 11	−4 ± 10	36	79 ± 15	−3 ± 16	40	71 ± 22	−9 ± 23	62
d/a flow ratio (–)	0.60 ± 0.05	−0.06 ± 0.07	22	0.75 ± 0.11	0.07 ± 0.23	61	0.62 ± 0.11	−0.06 ± 0.06	18

Data are presented as mean ± standard deviation. *aAo* Ascending aorta, *CO* Cardiac output, *CR* Coefficient of repeatability, calculated as 1.96 times the standard deviation of the differences between repeated measurements and expressed as a percentage of the group mean value, *dAo* Descending aorta, *EDV* End-diastolic volume, *EF* Ejection fraction, *ESV* End-systolic volume, *LV* Left ventricle, *RV* Right ventricle, *SV* Stroke volume

## Discussion

This work established the feasibility of biventricular volumetry with real-time cine MRI at 3 Tesla in healthy volunteers during in-magnet moderate- and vigorous-intensity bicycling exercise. We demonstrated that quantitative estimates of LV and RV function can be obtained during physical exercise at heart rates exceeding 140 beats/min. The inotropic effect of augmented myocardial contractility is evidenced by the consistent decrease in ESV for both ventricles with increasing exercise intensity, to effect a near tripling of CO at vigorous-intensity exercise. Moreover, we estimated that measurement precision will be sufficient to detect with 95% confidence any pathophysiological differences greater than 40% in LV or RV SV during vigorous exercise between two separate exercise MRI stress tests performed by a single subject. While this precision is similar to that of measurements at rest, clinical applications of exercise MRI stress testing for evaluating an individual patient’s cardiac performance in the context of disease progression monitoring and treatment efficacy testing will benefit from further improvement of measurement precision. 

Thorough repeatability assessments of exercise MR stress testing that cover the full protocol from subject positioning to quantitative data analyses are scarce. Lurz et al. [9] pooled CO measured during two steady-state exercise intensities to report an inter-session repeatability coefficient of approximately 10% for both ventricles, similar to the >10% inter-session repeatability coefficient for the cardiac index (i.e., CO normalized to body surface area) reported later for immediate post-exercise real-time imaging [11]. La Gerche et al. [17] found an excellent correlation between pooled CO measured at rest and three steady-state exercise intensities during repeated exercise protocols separated by 1 h of rest. Although the measurement precision of CO or cardiac index can be informative, other volumetric parameters may be more relevant in the context of diagnostic exercise MRI stress testing, e.g., RV ESV in athletes with arrhythmias [32] or LV and RV EDV in patients with repaired Tetralogy of Fallot [16]. Ghanbari et al. [12] investigated immediate post-exercise volumetric parameters other than CO by including LV and RV EDV and ESV in their analyses of healthy volunteers. Remarkably, that study reported extremely narrow (i.e., <10 mL) inter-session 95% confidence intervals for measurements at 3 Tesla that were conducted 1 year apart [12]. Volumetry performed during in-magnet exercise at 1.5 Tesla achieved repeatability coefficients of 11% (LV EDV, indexed to body surface area) to 47% (RV ESV, indexed) in healthy volunteers [19]. Notably, Chandrasekaran et al. [20] also found that measurement repeatability during bicycling exercise at 3 Tesla was poorest for RV ESV. In the present work, we provide a comprehensive evaluation of exercise MRI stress testing measurement precision using Bland–Altman analyses of inter-session measurement repeatability. In keeping with previous work, our precision for measuring RV ESV during vigorous exercise was the poorest of all volumetric and functional parameters. Precision for other parameters was <20% up to >40%, which is better than or similar to VO_2_max measurement repeatability with CPET [33, 34].

Resting-state validations of LV and RV EDV and ESV quantifications based on real-time cine MRI against conventional ECG-gated cine MRI revealed that real-time cine MRI underestimates SV by approximately 13%, which is mostly mediated by a bias in EDV quantification. We attribute this difference to the necessity of retrospectively identifying the end-diastolic and end-systolic phases *per slice* for real-time cine MRI, whereas particularly the end-diastolic phase is well defined for *all slices* in conventional ECG-gated cine MRI through the ECG *R*-wave that triggers image acquisition. Any deviation from the actual end-diastolic phase would lead to an underestimation of EDV. Consequently, SV will also be underestimated, which can be further affected by any deviations in end-systolic phase identification. Although moderate, this effect could impact the accuracy of the SV measured during exercise, where elevated heart rates may compromise the correct identification of end-diastolic and end-systolic phases. Acquisitions at even higher temporal resolution may mitigate this issue [6, 8], although further acceleration with parallel imaging will increase spatial blurring [23]. In addition, in a trade-off for higher temporal resolution, slice thickness and in-plane resolution were coarser for real-time cine MRI than for ECG-gated cine MRI, which may have negatively affected real-time cine MRI volumetry accuracy. Nonetheless, we found a progressive and consistent increase in SV with higher exercise intensities, which was quantitatively validated by 2D PC-MRI measurements of aortic blood flow. Note that coronary blood flow, while part of LV SV, is not captured by blood flow measurements of the aorta. Coronary blood flow constitutes a small proportion of LV SV (<5%), and increases linearly with heart rate during dynamic exercise [35]. Here, we did not detect such coronary blood flow-mediated underestimation of 2D PC-MRI relative to real-time cine MRI-based estimates of LV SV.

The term “exercise MRI” is commonly used to describe MRI assessments of the acute effects of physical activity. Yet, in practice, many studies acquire data only immediately *after* exercise, rather than during exercise [11–14]. While this approach helps reduce motion artifacts and avoid gating issues due to ECG-signal distortions, data are essentially collected during early recovery when heart rate may drop rapidly [36] and any ischemia resolves progressively [37]. In contrast, we acquired data *during* uninterrupted steady-state bicycle exercise using real-time cine MRI [9, 17, 18, 20]. Given the multitude of short-axis slices needed to achieve whole-heart coverage and the large number of real-time images per slice needed to ensure capturing the required cardiac and respiratory phases for each slice, the resultant stack contains many images making physiological frame sorting a time-consuming and labor-intensive task [17], even if respiratory motion is ignored [11]. More automated approaches have been developed, including principal component analysis of image signal intensities [38] and image-informed extraction of respiratory motion for quantification of LV function [18]. Similar to Edlund et al. [18], we used signal intensity periodicity to capture the respiratory cycle for retrospective frame sorting. Note that while manual input is still needed and that such an approach may remain a time-consuming task, the procedure is essentially similar to what is needed for estimating the end-expiratory pulmonary capillary wedge pressure from respiratory pressure swings during exercise right heart catheterization [39, 40], or the selection of relevant frames after completion of an exercise echocardiography stress test [37].

Exercise right heart catheterization is the current gold standard for diagnosing HFpEF [3]. With increasing research interest and development efforts [6], exercise MRI stress testing may become a noninvasive alternative to such invasive stress testing in patients with unexplained exertional dyspnea and fatigue [4]. By quantifying chamber volumes and stroke volumes, and potentially more innovative markers of systolic and diastolic function (e.g., strains or strain rates) during exercise, the relevance of EF [41, 42] will likely become less important for characterizing heart failure [3]. Future work may focus on achieving a higher temporal resolution through further parallel imaging acceleration [23] or retrospective binning of radial sampling trajectories [43], aimed at increasing accuracy and precision of biventricular volumetry or strain [8] quantifications. We used a custom-built 72-channel receiver coil array to increase the capacity for parallel imaging acceleration, which may limit the widespread applicability of this particular approach. We anticipate that machine learning-based reconstructions and advanced compressed-sensing methods [44] will offer the potential for achieving similar acceleration factors with commercially available receiver coil arrays.

In conclusion, we established an exercise MRI stress testing protocol for quantifying biventricular volumes and function during moderate- and vigorous-intensity steady-state bicycling exercise using real-time cine MRI at 3 Tesla. Estimates based on real-time MRI were validated against measurements with conventional ECG-gated cine MRI, as well as with 2D PC-MRI of the forward blood flow through the ascending aorta. This approach allows for noninvasive quantitative evaluations of the functional performance of both ventricles during physical exercise, at a measurement precision that is similar to other modalities of functional exercise stress testing (CPET, echocardiography) currently used in the (sports) cardiology workflow. As such, our work will contribute to consolidating exercise MR stress testing as a valuable tool for improving our understanding of human exercise physiology and exercise intolerance, as well as for diagnosing and risk stratification of heart failure and other multifactorial diseases.

## Supplementary Information

Below is the link to the electronic supplementary material.

Supplementary file1 (GIF 4849 KB)

Supplementary file2 (GIF 4278 KB)

Supplementary file3 (GIF 3942 KB)

Supplementary file4 (GIF 4952 KB)

Supplementary file5 (GIF 5285 KB)

Supplementary file 6 (GIF 4603 KB)

Supplementary file 7 (GIF 4551 KB)

Supplementary file 8 (GIF 5140 KB)

Supplementary file 9 (GIF 6633 KB)

Supplementary file 10 (GIF 564 KB)

Supplementary file 11 (GIF 676 KB)

Supplementary file 12 (GIF 732 KB)

Supplementary file13 (PDF 90 KB)

## Data Availability

The imaging data that support the findings of this study are available from the corresponding author upon reasonable request. Data are located in controlled-access data storage facilities at the Amsterdam University Medical Center.
