# Supplementary figures and images for: Exercise MRI stress testing of the human heart at 3 Tesla: measurement precision of biventricular function and aortic blood flow during steady-state bicycling exercise
Source: MAGMA. 2025 Dec 4;39(2):187–200. doi: 10.1007/s10334-025-01304-9 (PMC13078410; doi:10.1007/s10334-025-01304-9)

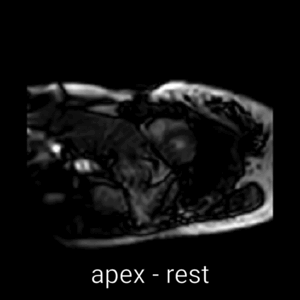

Supplement: Supplementary file 1 — Supplementary file1 (GIF 4849 KB) [file 10334_2025_1304_MOESM1_ESM.gif]

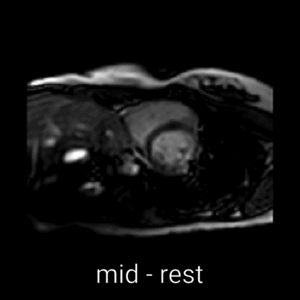

Supplement: Supplementary file 2 — Supplementary file2 (GIF 4278 KB) [file 10334_2025_1304_MOESM2_ESM.gif]

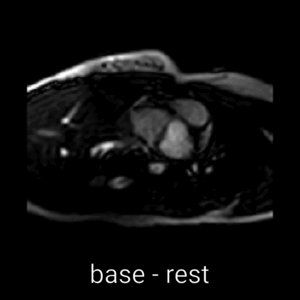

Supplement: Supplementary file 3 — Supplementary file3 (GIF 3942 KB) [file 10334_2025_1304_MOESM3_ESM.gif]

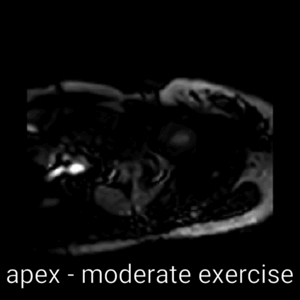

Supplement: Supplementary file 4 — Supplementary file4 (GIF 4952 KB) [file 10334_2025_1304_MOESM4_ESM.gif]

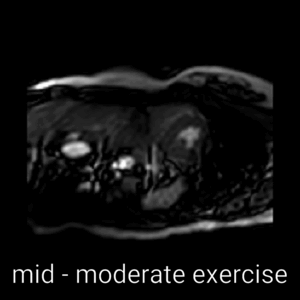

Supplement: Supplementary file 5 — Supplementary file5 (GIF 5285 KB) [file 10334_2025_1304_MOESM5_ESM.gif]

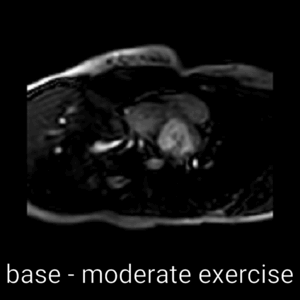

Supplement: Supplementary file 6 — (GIF 4603 KB) [file 10334_2025_1304_MOESM6_ESM.gif]

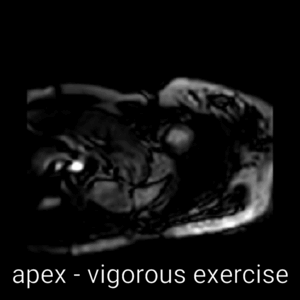

Supplement: Supplementary file 7 — (GIF 4551 KB) [file 10334_2025_1304_MOESM7_ESM.gif]

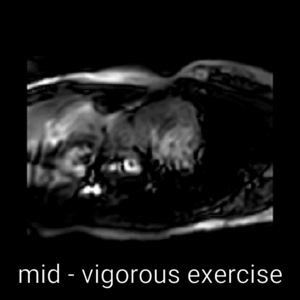

Supplement: Supplementary file 8 — (GIF 5140 KB) [file 10334_2025_1304_MOESM8_ESM.gif]

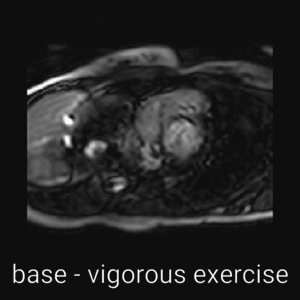

Supplement: Supplementary file 9 — (GIF 6633 KB) [file 10334_2025_1304_MOESM9_ESM.gif]

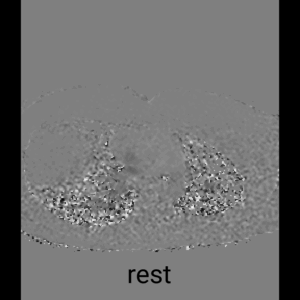

Supplement: Supplementary file 10 — (GIF 564 KB) [file 10334_2025_1304_MOESM10_ESM.gif]

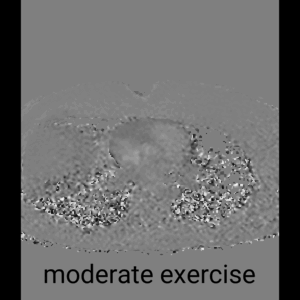

Supplement: Supplementary file 11 — (GIF 676 KB) [file 10334_2025_1304_MOESM11_ESM.gif]

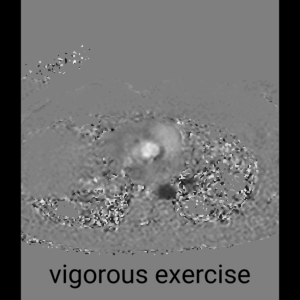

Supplement: Supplementary file 12 — (GIF 732 KB) [file 10334_2025_1304_MOESM12_ESM.gif]
