# Supplementary material for: Exercise MRI stress testing of the human heart at 3 Tesla: measurement precision of biventricular function and aortic blood flow during steady-state bicycling exercise
Source: MAGMA. 2025 Dec 4;39(2):187–200. doi: 10.1007/s10334-025-01304-9 (PMC13078410; doi:10.1007/s10334-025-01304-9)
Supplement: Supplementary file 13 — Supplementary file13 (PDF 90 KB) [file 10334_2025_1304_MOESM13_ESM.pdf]

Supplementary Information

Exercise MRI stress testing of the human heart at 3 Tesla: measurement precision of biventricular function and aortic blood flow during steady-state bicycling exercise

Hugo Klarenberg, Martijn Froeling, Tim Leiner, Hildo J. Lamb, S. Matthijs Boekholdt, Harald T. Jørstad, Gustav J. Strijkers, Adrianus J. Bakermans.

Supplemental Table 1. 95% confidence intervals for Bland-Altman analyses of inter-session measurement repeatability of exercise MRI stress testing in n = 6 volunteers.

	**rest**			**moderate exercise**			**vigorous exercise**		
	lower	upper	bias	lower	upper	bias	lower	upper	bias
LV EDV [mL]	-45; -14	1; 32	-15; 1	-134; -31	18; 121	-32; 20	-79; -25	0; 54	-26; 1
LV ESV [mL]	-79; -16	14; 76	-17; 15	-59; -9	15; 65	-10; 15	-81; -30	-5; 46	-31; -5
LV SV [mL]	-94; -23	12; 83	-24; 13	-154; -37	19; 136	-39; 21	-110; -17	27; 120	-18; 29
LV EF [%]	-51; -11	8; 48	-12; 9	-47; -10	7; 43	-11; 7	-41; 0	19; 60	-1; 20
LV CO [L/min]	-7.9; -1.8	1.1; 7.2	-1.9; 1.2	-16.9; -4.0	2.2; 15.1	-4.2; 2.4	-18.0; -2.9	4.3; 19.4	-3.1; 4.6
RV EDV [mL]	-62; -8	18; 71	-9; 18	-125; -15	38; 148	-17; 39	-98; -13	28; 113	-14; 29
RV ESV [mL]	-75; -5	29; 99	-6; 30	-32; 7	25; 63	6; 26	-100; -20	18; 98	-22; 19
RV SV [mL]	-92; -24	9; 77	-25; 10	-151; -33	24; 142	-35; 26	-100; -12	30; 118	-14; 31
RV EF [%]	-54; -15	3; 41	-19; 4	-50; -15	-2; 38	-15; 3	-47; -5	15; 56	-6; 15
RV CO [L/min]	-6.1; -1.6	0.6; 5.2	-1.6; 0.7	-16.0; -3.5	2.6; 15.1	-3.6; 2.8	-15.6; -2.0	4.5; 18.1	-2.2; 4.7
aAo flow [mL]	-79; -13	19; 85	-14; 20	-76; -25	-1; 49	-26; 0	-227; -33	14; 209	-54; 36
dAo flow [mL]	-57; -14	6; 49	-15; 7	-85; -19	13; 79	-19; 14	-183; -27	10; 166	-45; 27
dAo/aAo [-]	-0.4; -0.1	0.0; 0.3	-0.1; 0.0	-1.1; -0.2	0.3; 1.3	-0.2; 0.3	-0.5; -0.1	-0.0; 0.4	-0.2; 0.0

Data are presented as 95% confidence interval ranges for upper limit (upper), lower limit (lower), and bias (i.e., mean difference between consecutive sessions). aAo, ascending aorta; CO, cardiac output; dAo, descending aorta; EDV, end-diastolic volume; EF, ejection fraction; ESV, end-systolic volume; LV, left ventricle; RV, right ventricle; SV, stroke volume.

Supplemental Table 2. Intraclass correlation coefficients (ICC) for inter-session measurement repeatability of exercise MRI stress testing in n = 6 volunteers.

	**rest**			**moderate exercise**			**vigorous exercise**		
	ICC	95% CI		ICC	95% CI		ICC	95% CI	
LV EDV [mL]	0.892	0.298	0.983	0.531	-0.210	0.913	0.796	0.055	0.966
LV ESV [mL]	0	-1.143	0.663	0.264	-0.758	0.852	0.188	-0.155	0.670
LV SV [mL]	0.482	-0.433	0.898	0.163	-0.596	0.815	0.478	-0.519	0.903
LV EF [%]	0	-1.298	0.273	0	-1.168	0.443	0	-0.392	0.680
LV CO [L/min]	0.273	-0.738	0.854	0.104	-0.821	0.802	0.246	-0.800	0.850
RV EDV [mL]	0.768	0.281	0.958	0.591	-0.211	0.918	0.845	0.318	0.972
RV ESV [mL]	0.401	-0.259	0.859	0.582	-0.112	0.948	0.587	-0.458	0.932
RV SV [mL]	0.429	-0.397	0.875	0.248	-0.825	0.853	0.485	-0.189	0.892
RV EF [%]	0.331	-0.224	0.831	0.228	-0.340	0.781	0	-0.782	0.591
RV CO [L/min]	0.435	-0.418	0.880	0.252	-0.829	0.855	0.421	-0.463	0.879
aAo flow [mL]	0.565	-0.418	0.923	0.470	-0.171	0.871	0.697	-0.563	0.974
dAo flow [mL]	0.649	0.040	0.933	0.598	-0.387	0.931	0.603	-0.660	0.963
dAo/aAo [-]	0.346	-0.146	0.842	0	-0.909	0.647	0.754	-0.090	0.983

Intraclass correlation coefficient (ICC) estimates and 95% confidence intervals (CI) are based on a single-measurement absolute-agreement two-way mixed-effects model. aAo, ascending aorta; CO, cardiac output; dAo, descending aorta; EDV, end-diastolic volume; EF, ejection fraction; ESV, end-systolic volume; LV, left ventricle; RV, right ventricle; SV, stroke volume.
